# Brain abnormalities, neurodegeneration, and dysosteosclerosis (BANDDOS): new cases, systematic literature review, and associations with CSF1R-ALSP

**DOI:** 10.1186/s13023-023-02772-9

**Published:** 2023-06-22

**Authors:** Jarosław Dulski, Josiane Souza, Mara Lúcia Santos, Zbigniew K. Wszolek

**Affiliations:** 1 Department of Neurology, Mayo Clinic, 4500 San Pablo Rd S, Jacksonville, FL 32224 USA; 2 Division of Neurological and Psychiatric Nursing, Faculty of Health Sciences, Medical University of Gdansk, Gdansk, 80-211 Poland; 3 Neurology Department, St Adalbert Hospital, Copernicus PL Ltd, Gdansk, 80-462 Poland; 4 School of Medicine, Pontificia Universidade Católica do Paraná (PUCPR), Curitiba, Paraná 80215-901 Brazil; 5 Department of Genetics, Hospital Infantil Pequeno Príncipe, Curitiba, Paraná 80240-020 Brazil; 6 Department of Neurology, Hospital Infantil Pequeno Príncipe, Curitiba, Paraná 80240-020 Brazil

**Keywords:** CSF1R, Microglia, Leukoencephalopathy, Axonal spheroids

## Abstract

*CSF1R* mutations cause autosomal-dominant *CSF1R*-related leukoencephalopathy with axonal spheroids and pigmented glia (*CSF1R*-ALSP) and autosomal-recessive brain abnormalities, neurodegeneration, and dysosteosclerosis (BANDDOS). The former is increasingly recognized, and disease-modifying therapy was introduced; however, literature is scarce on the latter. This review analyzes BANDDOS and discusses similarities and differences with *CSF1R*-ALSP.

We systematically retrieved and analyzed the clinical, genetic, radiological, and pathological data on the previously reported and our cases with BANDDOS. We identified 19 patients with BANDDOS (literature search according to the PRISMA 2020 guidelines: n = 16, our material: n = 3). We found 11 *CSF1R* mutations, including splicing (n = 3), missense (n = 3), nonsense (n = 2), and intronic (n = 2) variants and one inframe deletion. All mutations disrupted the tyrosine kinase domain or resulted in nonsense-mediated mRNA decay. The material is heterogenous, and the presented information refers to the number of patients with sufficient data on specific symptoms, results, or performed procedures. The first symptoms occurred in the perinatal period (n = 5), infancy (n = 2), childhood (n = 5), and adulthood (n = 1). Dysmorphic features were present in 7/17 cases. Neurological symptoms included speech disturbances (n = 13/15), cognitive decline (n = 12/14), spasticity/rigidity (n = 12/15), hyperactive tendon reflex (n = 11/14), pathological reflexes (n = 8/11), seizures (n = 9/16), dysphagia (n = 9/12), developmental delay (n = 7/14), infantile hypotonia (n = 3/11), and optic nerve atrophy (n = 2/7). Skeletal deformities were observed in 13/17 cases and fell within the dysosteosclerosis – Pyle disease spectrum. Brain abnormalities included white matter changes (n = 19/19), calcifications (n = 15/18), agenesis of corpus callosum (n = 12/16), ventriculomegaly (n = 13/19), Dandy-Walker complex (n = 7/19), and cortical abnormalities (n = 4/10). Three patients died in infancy, two in childhood, and one case at unspecified age. A single brain autopsy evidenced multiple brain anomalies, absence of corpus callosum, absence of microglia, severe white matter atrophy with axonal spheroids, gliosis, and numerous dystrophic calcifications.

In conclusion, BANDDOS presents in the perinatal period or infancy and has a devastating course with congenital brain abnormalities, developmental delay, neurological deficits, osteopetrosis, and dysmorphic features. There is a significant overlap in the clinical, radiological, and neuropathological aspects between BANDDOS and *CSF1R*-ALSP. As both disorders are on the same continuum, there is a window of opportunity to apply available therapy in *CSF1R*-ALSP to BANDDOS.

## Introduction

Mutations in the *colony-stimulating factor-1 receptor (CSF1R)* gene may account for up to 25% of adult-onset leukoencephalopathies [1, 2]. Most of the previously reported *CSF1R* mutation carriers had only one mutant allele and presented with an autosomal-dominant neurodegenerative disorder characterized by neuropsychiatric and motor symptoms, white matter lesions on magnetic resonance imaging (MRI), brain calcifications with stepping stone appearance on computed tomography, axonal spheroids and pigmented glia on neuropathological examination [3, 4]. The disease was previously known as hereditary diffuse leukoencephalopathy with spheroids (HDLS) or pigmentary orthochromatic leukodystrophy, but the expanding knowledge of leukoencephalopathies led to the new classification, and it was named adult-onset leukoencephalopathy with axonal spheroids and pigmented glia (ALSP) [1, 2, 5, 6]. Most cases of ALSP are due to the *CSF1R* mutations; however, *AARS2* mutations were reported in CSF1R-negative ALSP, a single Swedish HDLS family was found to carry an *AARS1* mutation, and ALSP without *CSF1R, AARS1* or *AARS2* mutation was reported [7–9]. As of January 2023, approximately 300 cases of *CSF1R*-ALSP were reported, but with genetic testing available commercially, the disease is increasingly recognized, and at present, the prevalence is estimated at 30–75 cases/million [1].

Furthermore, few case reports have been published with patients carrying two mutant *CSF1R* alleles presenting with brain abnormalities, neurodegeneration, and dysosteosclerosis (BANDDOS), and a new entity has been recognized (MIM#618,476) [10]. The literature on BANDDOS is scarce, and the clinical presentation and radiological and neuropathological features are yet to be elucidated. Disease-modifying treatment is available for the *CSF1R*-ALSP, and an interventional clinical trial is underway (NCT05677659). As both BANDDOS and *CSF1R*-ALSP share a genetic basis, treatment of the latter could be potentially translated to the former. A better understanding of the BANDDOS would also benefit *CSF1R*-ALSP, which pathomechanism remains not fully understood.

In this paper, we add to the growing literature on the pathogenicity of *CSF1R* mutations and their genotype-phenotype associations by reviewing the clinical, genetic, radiological, and neuropathological features of BANDDOS. We analyzed three new BANDDOS patients from a Brazilian family and the previously published cases in the literature.

## Methods

We collected the clinical, genetic, and radiological data on the family diagnosed with BANDDOS observed at the Hospital Pequeno Principe, Curitiba, Paraná, Brazil. Each individual was repeatedly evaluated by a multidisciplinary team, including geneticist, neurologist, psychologist, and radiologist. Genetic testing was performed in 5 individuals, including whole-exome sequencing (WES) in the proband and targeted sequencing in four others. WES was performed using an Agilent v5 SureSelect capture kit and Illumina HiSeq 2500 sequencing technology. Reads were aligned to a reference sequence (GRCh38), and sequence changes were identified and interpreted in the context of relevant transcripts. Targeted sequencing was performed with next-generation sequencing (NGS) technology. Brain 1.5 Tesla MRI was performed in 3 cases.

Next, we searched the MEDLINE, PubMed, Scopus, and Web of Science databases for papers on BANDDOS published until December 16, 2022. The literature search was conducted according to the PRISMA 2020 guidelines [11]. We applied the following search terms: “BANDDOS”, “CSF1R” and “homozygous”, “CSF1R” and “autosomal recessive”. We screened the titles and abstracts of the papers to check if they were relevant to the review. We searched the reference list of the relevant manuscripts and websites to identify other papers pertinent to the review.

Lastly, we extracted in a structured manner the data on the Brazilian family with BANDDOS and previously published cases identified in the literature. We retrieved the information on *CSF1R* mutation, demographics (sex, ethnicity, consanguinity of the parents, gestational age, birth weight, birth length, age of onset, follow-up duration, weight, and height at the last follow-up, age of death), brain autopsy, dysmorphic features, first symptoms, neurological status (infantile hypotonia, developmental delay, cognitive decline, seizures, optic nerve atrophy, dysphagia, speech disturbances, rigid-spasticity, hyperactive tendon reflexes, pathological reflexes), skeletal system symptoms (bone and tooth abnormalities), and brain imaging (Dandy-Walker malformation, ventriculomegaly, calcifications, agenesis of corpus callosum, white matter changes and cortical abnormalities).

## Results

**Fig. 1:**
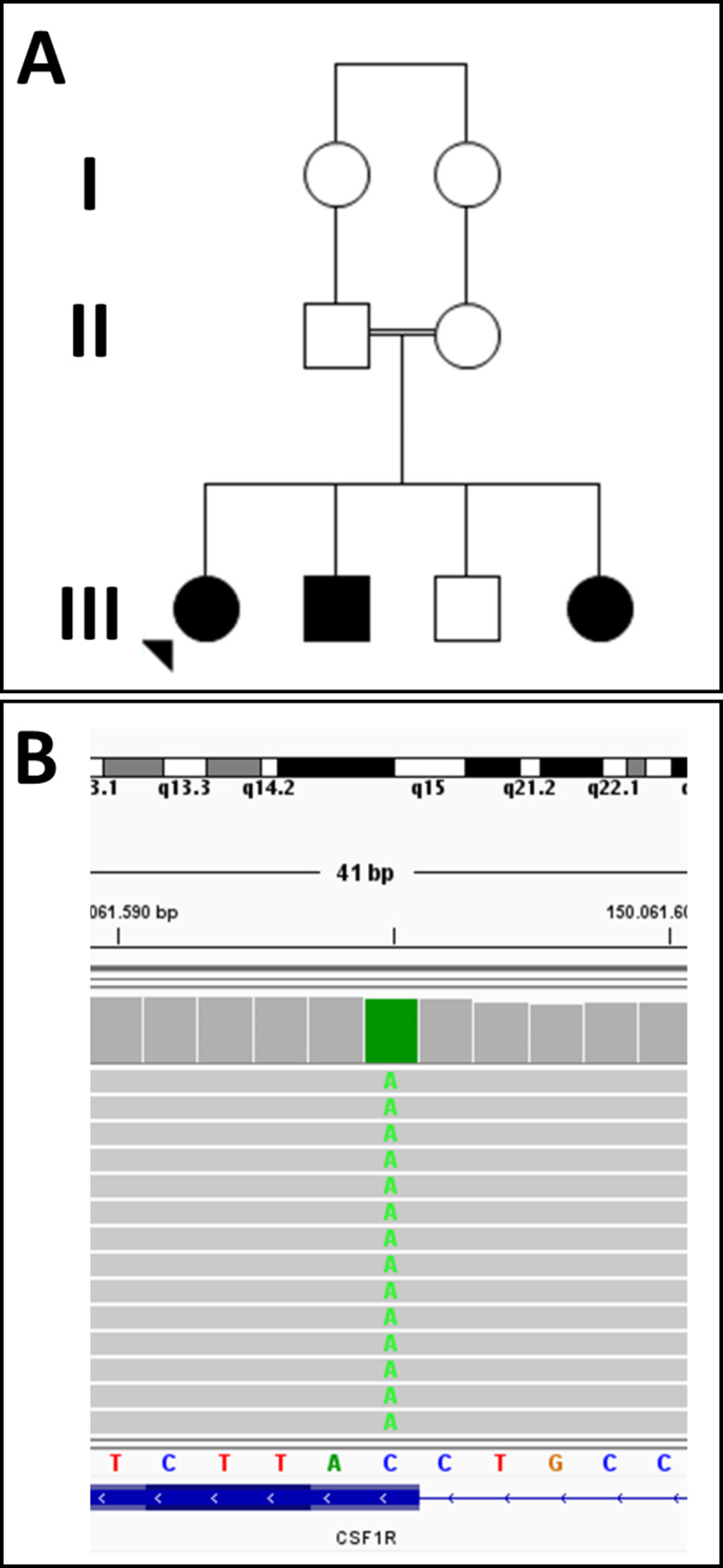
(**A**) Pedigree. For family pedigree, standard pedigree symbols are used; arrow indicates the proband; circles indicate females; squares indicate males; black symbols indicate individuals diagnosed with brain abnormalities, neurodegeneration, and dysosteosclerosis (BANDDOS). (**B**) The Integrative Genomics Viewer snapshot displaying the newly identified *CSF1R* mutation.

We identified three unreported siblings with homozygous *CSF1R* c.1754G > T (Gly585Val) mutations from Curitiba, Paraná, Brazil (Fig. 1). They were born to consanguineous parents (first cousins) and did not display dysmorphic features, bone abnormalities, or developmental delay in the first few years of their life. The III-1 developed cognitive regression and speech disturbances at 10 years, followed by seizures, dysphagia, and spastic tetraparesis over the following six years. The III-2 developed neuropsychiatric symptoms at the age of 15 years, whereas III-4 remained asymptomatic at the age of 9 years. All three had white matter changes on neuroimaging with MRI. The III-3 and II-1 were heterozygous for the Gly585Val mutation and remained asymptomatic, the II-2 refused genetic testing. *In silico* analysis predicted the newly identified mutation to be pathogenic (Combined Annotation Dependent Depletion score of 35) and it was likely pathogenic (PM1, PM2, PM5, PP2, PP3, PP4) according to the guidelines of the American College of Medical Genetics and Genomics and the Association for Molecular Pathology [12].

**Fig. 2:**
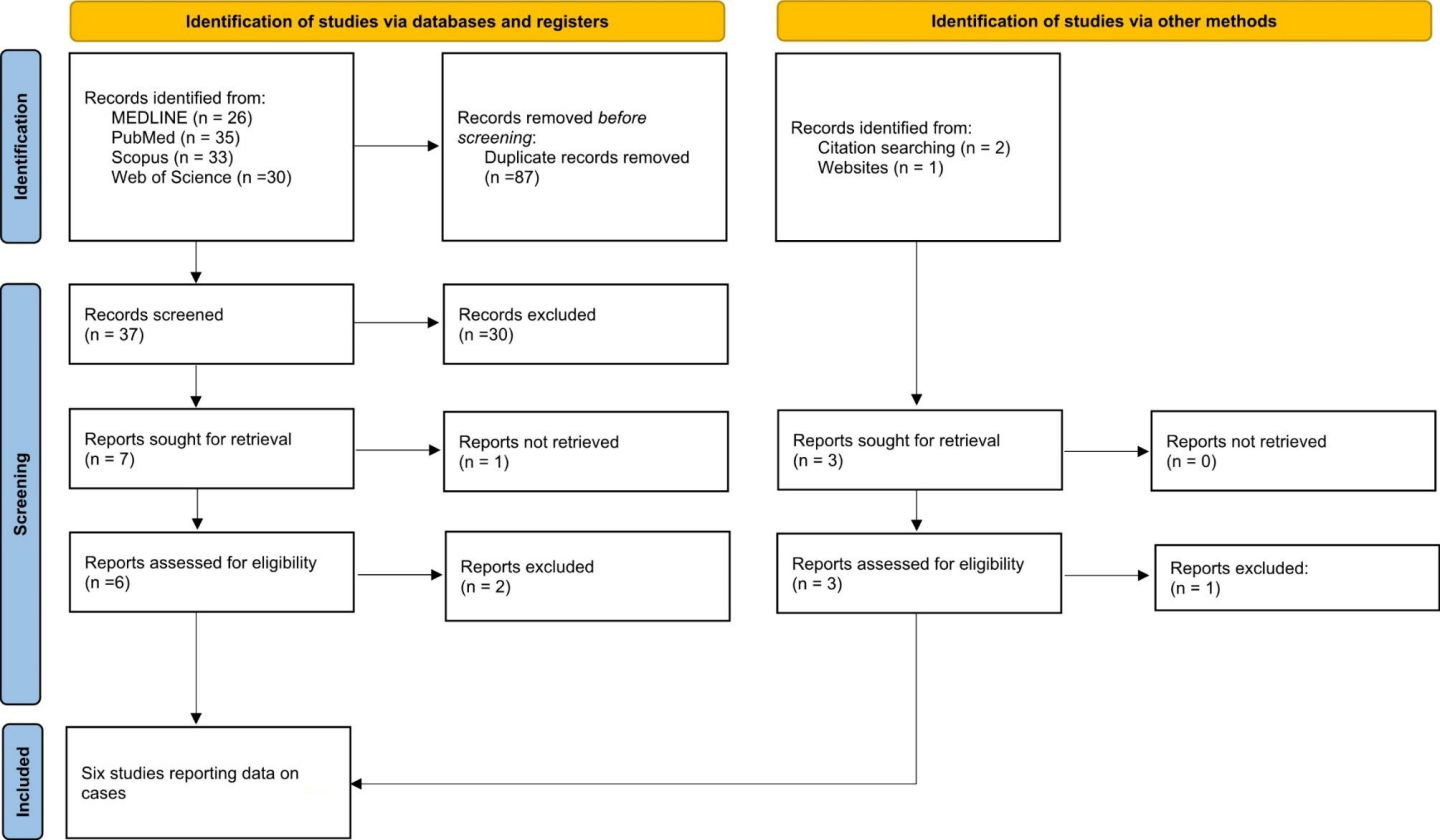
PRISMA 2020 flow diagram for new systematic reviews, which included searches of databases, registers, and other sources *Consider, if feasible to do so, reporting the number of records identified from each database or register searched (rather than the total number across all databases/registers) **If automation tools were used, indicate how many records were excluded by a human and how many were excluded by automation tools *From*: Page MJ, McKenzie JE, Bossuyt PM, Boutron I, Hoffmann TC, Mulrow CD, et al. The PRISMA 2020 statement: an updated guideline for reporting systematic reviews. BMJ 2021;372:n71. doi: 10.1136/bmj.n71. For more information, visit: http://www.prisma-statement.org/

Literature search for papers on BANDDOS yielded 124 records. After removing the duplicates (n = 87), we screened 37 papers, and subsequently excluded 30 that were not pertinent to the topic. We were able to retrieve 6/7 of the remaining manuscripts and assessed 4/6 to be relevant to the topic. We identified 3 additional papers on BANDDOS through citation and website searching, of which 2 were pertinent to the topic and were included in the review. The PRISMA 2020 flow diagram for the literature review on BANDDOS is presented in Fig. 2.

**Fig. 3:**
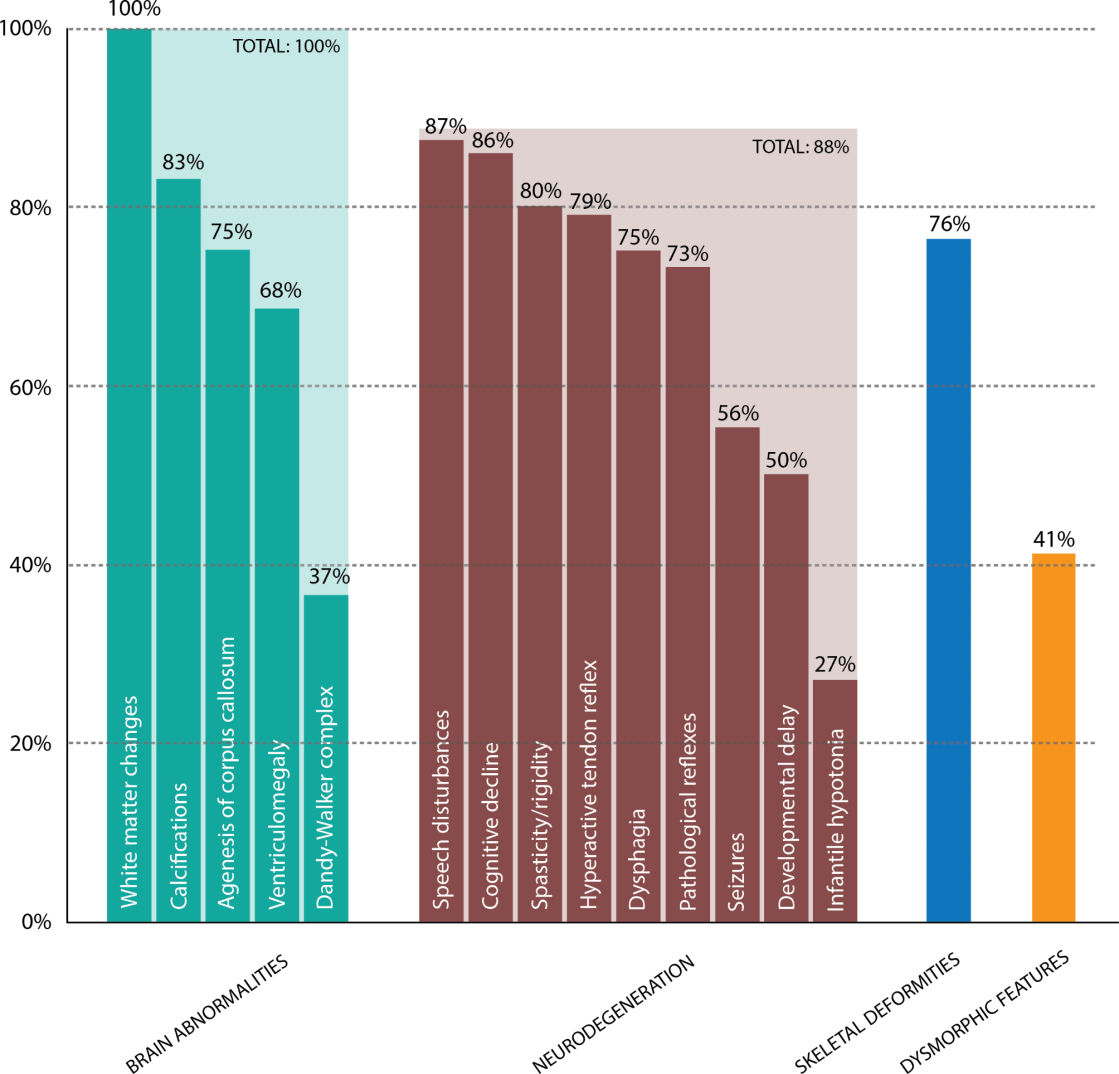
The chart presents the main features of brain abnormalities, neurodegeneration, and dysosteosclerosis (BANDDOS). The y-axis represents the frequency; the x-axis represents the features

The demographics and CSF1R mutations of the patients with BANDDOS are presented in Table 1. The material is heterogenous, and the presented information refers to the number of patients with sufficient data regarding the specific symptoms, results, or performed procedures. Figure 3 presents the chart with the main features of BANDDOS and their frequency.

**Table 1:** The demographics and *CSF1R* variants of the patients with BANDDOS

No	Paper	CSF1R mutations	Sex	Ethnicity	Consanguinity of the parents	Gestational age (weeks)	Birth weight (g)	Birth length (cm)	Age of onset	Follow-up duration (years)	Age at last follow-up (years)	Weight at last follow-up (kg)	Height at last follow-up (cm)	Age of death	Brain autopsy
**1**	Monies et al. (2017)	*bi-allelic c.1620T > A	N/R	Arab	Yes	N/R	N/R	N/R	N/R	N/R	N/R	N/R	N/R	Infancy (details not reported)	No
**2**	Monies et al. (2017)	*bi-allelic c.1620T > A	N/R	Arab	Yes	N/R	N/R	N/R	N/R	N/R	N/R	N/R	N/R	Infancy (details not reported)	No
**3**	Guo et al. (2019)	Allele 1: c.395 C > T, Allele 2: c.1441 C > T	Male	Brazilian	No	Early term (37 weeks 5 days)	4270	50	Prenatal	5	4.8	Low (14.5 kg, 5–10 percentile)	Short (94.5 cm, Z-score − 2.8)	N/A	N/A
**4**	Guo et al. (2019)	Allele 1: c.1859-119G > A Allele 2: c.1879_1881del	Female	Japanese	No	Full term	N/R	N/R	28 years	9	37	Normal (42.8 kg)	Normal (152.7 cm)	N/A	N/A
**5**	Guo et al. (2019) & Helman et al. (2020)	bi-allelic c.1969 + 115_1969 + 116del	Female	Chaldean	Yes	Full term	N/R	N/R	N/R	N/R	23	Normal (45.9 kg)	Normal (153.2 cm)	N/A	N/A
**6**	Guo et al. (2019) & Helman et al. (2020)	bi-allelic c.1969 + 115_1969 + 116del	Male	Chaldean	Yes	Full term	3300	N/R	Perinatal	14	14	Normal (50 kg)	Short (148 cm)	N/A	N/A
**7**	Guo et al. (2019) & Helman et al. (2020)	bi-allelic c.1969 + 115_1969 + 116del	Female	Chaldean	Yes	N/R	N/R	N/R	Perinatal	N/R	N/R	N/R	N/R	N/A	N/A
**8**	Guo et al. (2019) & Helman et al. (2020)	bi-allelic c.1969 + 115_1969 + 116del	Female	Chaldean	Yes	N/R	N/R	N/R	N/R	N/R	N/R	N/R	N/R	N/A	N/R
**9**	Guo et al. (2019) & Helman et al. (2020)	bi-allelic c.1969 + 115_1969 + 116del	Female	Chaldean	Yes	N/R	N/R	N/R	N/R	N/R	N/R	N/R	N/R	Deceased (age not reported)	N/R
**10**	Oosterhof et al. (2019)	bi-allelic c.1754-1G > C	Male	Native American	Yes	Pre-term (35 weeks 4 days)	4106	N/R	Prenatal	1	0.8	N/R	N/R	10 months	Yes
**11**	Oosterhof et al. (2019)	bi-allelic c.1929 C > A	Male	Arab	Yes	N/R	N/R	N/R	12 years old	12	24	N/R	N/R	N/A	N/A
**12**	Tamhankar et al. (2020)	bi-allelic c.2498 C > T	Female	Indian	Yes	N/R	N/R	N/R	2 years old	8	12	N/R	N/R	12 years	N/R
**13**	Tamhankar et al. (2020)	bi-allelic c.2498 C > T	Male	Indian	Yes	N/R	N/R	N/R	4 years old	0.67	4.67	N/R	N/R	4 years 8 months	N/R
**14**	Kındıs et al. (2021)	bi-allelic c.2763 + 1G > T	Female	Turkish	Yes	Full term	3250	N/R	Infancy	10	10	N/R	N/R	N/A	N/A
**15**	Kındıs et al. (2021)	bi-allelic c.2763 + 1G > T	Male	Turkish	Yes	Full term	3600	N/R	Perinatal	7	7	Normal (28 kg)	Normal (116.5 cm)	N/A	N/A
**16**	Kındıs et al. (2021)	bi-allelic c.2763 + 1G > T	Female	Turkish	Yes	Full term	3500	N/R	Infancy	16	16	Normal (54 kg)	Normal (158 cm)	N/A	N/A
**17**	Our case	bi-allellic c.1754G > T	Female	Brazilian	Yes	Full term	3050	50	7 years old	11	18	50	152	N/A	N/A
**18**	Our case	bi-allellic c.1754G > T	Male	Brazilian	Yes	Full term	3500	50	15 years old	11	15	62	160	N/A	N/A
**19**	Our case	bi-allellic c.1754G > T	Female	Brazilian	Yes	Full term	3950	50	Asymptomatic	9	9	42	145	N/A	N/A

**Fig. 4:**
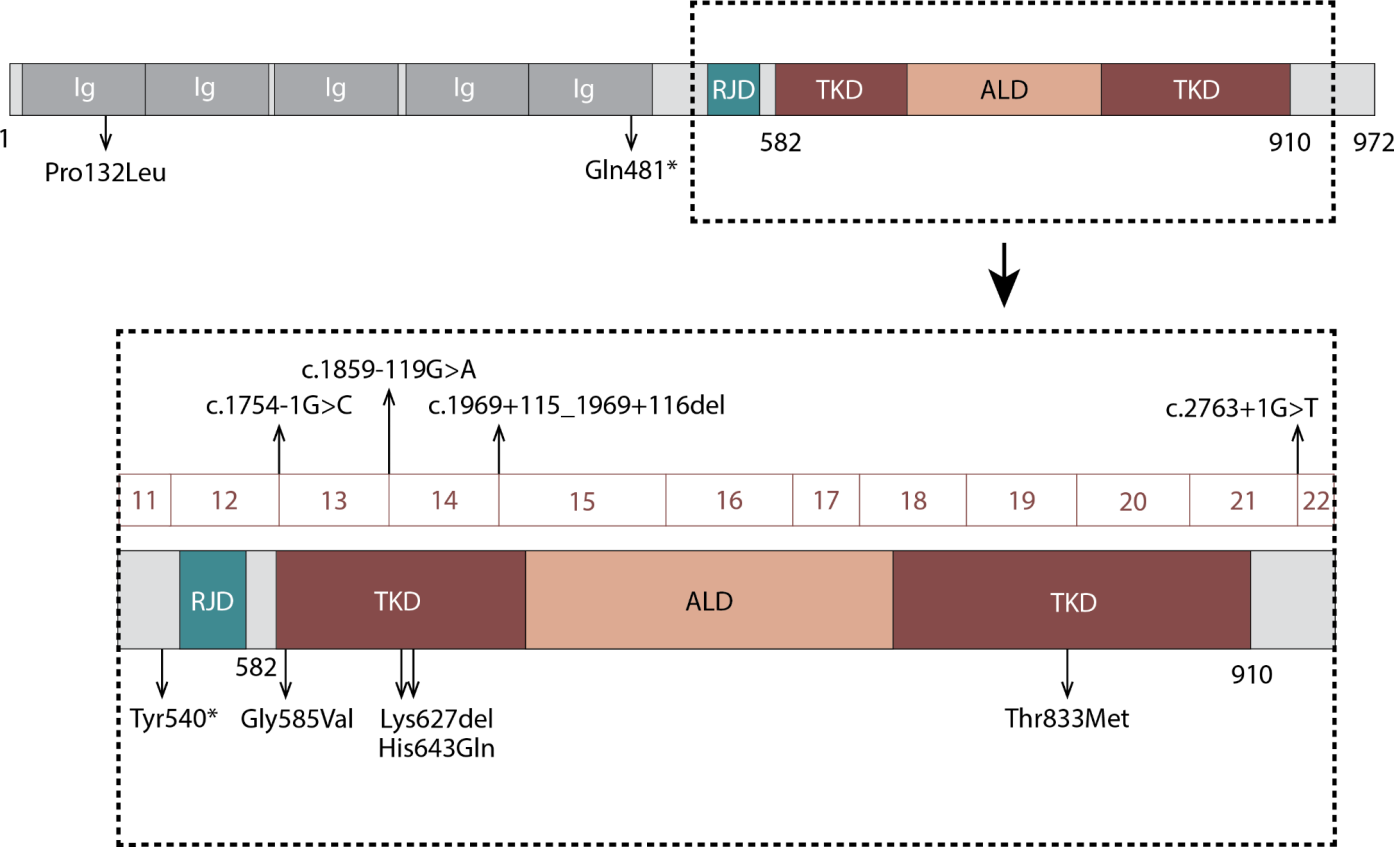
Schematic diagram of CSF1R gene and protein with mutations reported in BANDDOS. ALD - activation loop domain; Ig - immunoglobulin domain; RJD - regulatory juxtamembrane domain; TKD - tyrosine kinase domain

*Presumed (detected in a heterozygote state in parents); F = female; M = male; N/A = non-applicable; N/R = not reported;

We identified a total of 19 cases (10 females, 7 males and 2 unreported sex) diagnosed with BANDDOS, including three new cases from the Brazilian family and 16 cases through the literature search [10, 13–17]. Most cases (n = 17/19) were born of consanguineous parents and carried homozygous mutations (n = 17/19), whereas only two were compound heterozygotes born of unrelated parents. We found 11 different *CSF1R* mutations, including splicing variants (n = 3), missense (n = 3), nonsense (n = 2), and intronic (n = 2) variants, and one inframe deletion. Further analysis revealed that all mutations led to functionally deficient CSF1R protein with disrupted tyrosine kinase domain or nonsense-mediated mRNA decay. Figure 4 depicts *CSF1R* gene and protein with mutations reported in BANDDOS, and Table 2 provides detailed information on them.

**Table 2:** The *CSF1R* mutations reported in BANDDOS

Paper	HGVS c.	HGVS p.	Type	Result	CADD_phred	ACMG-AMP classification
Monies et al. (2017)	c.1620T > A	Tyr540*	Nonsense mutation (Premature stop codon)	Truncated CSF1R protein without intracellular part of the receptor	38	Pathogenic (PVS1, PM2, PP3, PP4, PP5)
Guo et al. (2019)	c.395 C > T	Pro132Leu	Missense variant	Functionally deficient CSF1R protein	25	Pathogenic (PS3, PM2, PM3, PP2, PP3, PP4, PP5)
Guo et al. (2019)	c.1441 C > T	Gln481*	Nonsense mutation (Premature stop codon)	Truncated CSF1R protein without intracellular part of the receptor	31	Pathogenic (PVS1, PS3, PM2, PP3, PP4, PP5)
Guo et al. (2019)	c.1859-119G > A	Ser620delins40	Intronic variant	Splicing mutation generating a novel cryptic splice acceptor site	13	Pathogenic (PS3, PM1, PM2, PM4, PP1, PP3, PP4, PP5)
Guo et al. (2019);	c.1879_1881del	Lys627del	Inframe deletion	In-frame deletion of lysine in the intracellular kinase domain of CSF1R causing functionally deficient CSF1R protein	Not available	Pathogenic (PS3, PM1, PM2, PM4. PP3, PP4, PP5)
Guo et al. (2019); Helman et al. (2020)	c.1969 + 115_1969 + 116del	Pro658Serfs*24	Intronic variant	Splicing mutation leading to the inclusion of the cryptic-exon, resulting in an in-frame stop codon, and nonsense-mediated mRNA decay	Not available	Pathogenic (PS3, PM1, PM2, PM4, PP3, PP4, PP5)
Oosterhof et al. (2019)	c.1754-1G > C	Gly585_Lys619delinsAla	Splicing mutation	Disruption of a splice acceptor site, leading to skipping of the amino acids 585–619 within the tyrosine kinase domain and production of an in-frame protein product	33	Likely Pathogenic (PM1, PM2, PM4, PP3, PP4, PP5)
Oosterhof et al. (2019)	c.1929 C > A	His643Gln	Missense variant	Functionally deficient CSF1R protein	4	Likely Pathogenic (PM1, PM2, PP1, PP2, PP4, PP5)
Tamhankar et al. (2020)	c.2498 C > T	Thr833Met	Missense variant	Functionally deficient CSF1R protein	29	Pathogenic (PS3, PM1, PM2, PP2, PP3, PP4, PP5)
Kındıs et al. (2021)	c.2763 + 1G > T	Not available	Splicing mutation	Aberrant splicing causing disruption of tyrosine kinase domain	34	*Likely Pathogenic (PM2, PM4, PP3, PP4, PP5)
Our cases	c.1754G > T	Gly585Val	Splicing mutation^#^	Disruption of a splice acceptor site resulting in disruption of tyrosine kinase domain	35	Likely Pathogenic (PM1, PM2, PM5, PP2, PP3, PP4)

*The authors concluded that the variant was pathogenic according to the ACMG-AMP criteria. ^#^Based on in silico models

ACMG-AMP = American College of Medical Genetics and Genomics and the Association for Molecular Pathology; CADD = Combined Annotation Dependent Depletion; HGVS = Human Genome Variation Society;

Patients were from different parts of the world and of various ethnic backgrounds, with Chaldean (n = 5), Brazilian (n = 4), Arab (n = 3), Turkish (n = 3), Indian (n = 2), Japanese, and Native American ancestry in individual cases. Most cases (9/11) were born full term, and only one was born prematurely. The mean weight and length at birth were 3600 g and 50 cm. Five cases became symptomatic before birth, 2 in infancy, and 5 in childhood, whereas only one showed first symptoms when adult. The mean follow-up duration was 9 years (8 months − 16 years). Dysmorphic features were present in 7 out of 17 cases, whereas weight and height were normal in 8/9 and 6/9, respectively, at the last evaluation at the mean age of 16 years (range 5–37 years).

Clinical characteristics of the patients with BANDDOS are presented in Table 3.

**Table 3:** Clinical characteristics of the patients with BANDDOS

No.	Dysmorphic features (n = 7/17)	First symptoms	Infantile hypotonia (n = 3/11)	Developmental delay (n = 7/14)	Cognitive decline (n = 12/14)	Seizure (n = 9/16)	Optic nerve atrophy (n = 2/7)	Dysphagia (n = 9/12)	Speech disturbances (n = 13/15)	Spasticity/rigidity (n = 12/15)	Hyperactive tendon reflex (n = 11/14)	Pathological reflexes (n = 8/11)	Bone abnormalities (n = 13/17)	Tooth (n = 1/7)
1	N/R	N/R	N/R	N/R	N/R	N/R	N/R	N/R	N/R	N/R	N/R	N/R	Osteopetrosis	N/R
2	N/R	N/R	N/R	N/R	N/R	N/R	N/R	N/R	N/R	N/R	N/R	N/R	Osteopetrosis	N/R
3	Long eyelashes, bilateral epicanthus, bulbous nose, sagging cheeks, tented upper lip, dysplastic ears, abnormal enamel in some of his teeth, narrow and bell-shaped thorax with pectus carinatum; joint restrictions at the elbows and ankle, and dorsal kyphosis	Multiple brain abnormalities, hypotonia and focal seizures in infancy	Yes	Yes, psychomotor retardation until 2.5 years old, than regression	Yes, early progressive decline	Yes	Yes	Yes	Yes, severe dysarhtria	Yes	Yes	N/R	Skeletal dysplasia (osteopetrosis spectrum) (dysosteosclerosis)	Abnormal enamel
4	Short limbs	Deterioration of vision due to the increased intra-crianial pressure	No	No	Yes, progressive decline in her 30s	No	Yes	No	Yes, progressive decline	Yes	Yes	Yes	Skeletal dysplasia (osteopetrosis spectrum) (dysosteosclerosis)	Normal
5	No	Developmental delay, intellectual disability	No	Yes, mild intellectual disability (IQ 73)	Yes, progressive decline	No	No	Yes	Yes, dysarthria	Yes	Yes	Yes	Skeletal dysplasia (osteopetrosis spectrum) (Pyle disease)	Normal
6	No	Hypotonia, seizures	Yes	Yes, intellectual disability (IQ 50)	Yes, progressive decline	Yes	No	Yes	Yes, dysarthria	Yes	Yes	Yes	Skeletal dysplasia (osteopetrosis spectrum) (Pyle disease)	Normal
7	No	N/R	N/R	N/R	Yes, progressive decline	Yes	N/R	Yes	Yes, dysarthria	Yes	Yes	Yes	Skeletal dysplasia (osteopetrosis spectrum) (Pyle disease)	N/R
8	No	N/R	N/R	N/R	Yes, progressive decline	Yes	N/R	Yes	Yes, dysarthria	Yes	Yes	Yes	Skeletal dysplasia (osteopetrosis spectrum) (Pyle disease)	N/R
9	No	N/R	N/R	N/R	Yes, progressive decline	Yes	N/R	Yes	Yes, dysarthria	Yes	Yes	Yes	Skeletal dysplasia (osteopetrosis spectrum) (Pyle disease)	N/R
10	Cranial asymmetry, flattened midface, depressed nasal bridge, deep palmar creases, bony prominences in the bilateral parietal skull	Diagnosed prenatally, born via primary cesarean section due to multiple fetal anomalies	Yes	Yes	N/R	Yes	N/R	Yes	N/R	Yes	No	N/R	Osteopetrosis	N/R
11	No	seizures, developmental regression	N/R	No	Yes, progressive decline since 12 years old	Yes	N/R	Yes	Yes, severe dysarhtria	N/R	Yes	N/R	No	N/R
12	Microcephaly, tall forehead, coarse facies, arched eyebrows, depressed nasal bridge, large protruding teeth and low set ears,	Encepalopathy	No	No	Yes, since 2 years old	N/R	N/R	N/R	Yes, since 2 years old	Yes	Yes	Yes	N/R	N/R
13	No	Walking on the toes	No	No	Yes	Yes	N/R	N/R	Yes, since 4 years old	Yes	N/R	N/R	N/R	N/R
14	Wide forehead, low posterior hairline, down slanting palpebral fissures, prominent nasal bridge, high-arched palate, and generalized joint laxity	Psychomotor develepmental delay	No	Yes	Yes, since 7 years old	No	N/R	N/R	Yes, dysarthria	Yes	Yes	No	Osteopetrosis, Erlenmeyer flask deformity	N/R
15	Dolichocephaly, hypertelorism, bilateral epicanthus	Respiratory distress after birth	N/R	Yes	N/R	No	N/R	N/R	Yes, dysarthria	No	N/R	N/R	Osteopetrosis	N/R
16	Mild ptosis, down slanting palpebral fissures, high arched palate, and prognathism	Psychomotor develepmental delay	N/R	Yes	N/R	No	N/R	N/R	N/R	N/R	N/R	N/R	Osteopetrosis, Erlenmeyer flask deformity	N/R
17	No	Gait change	No	No	Yes, since 10 years old	Yes	No	Yes	Yes, progressive decline	Yes	Yes	Yes	No	Normal
18	No	Psychiatric/depression	No	No	No	No	No	No	No	No	No	No	No	Normal
19	No	Asymptomatic	No	No	No	No	No	No	No	No	No	No	No	Normal

N/A = non-applicable; N/R = not reported;

Neurological symptoms were observed in most cases (n = 15/17), including speech disturbances (n = 13/15), cognitive decline (n = 12/14), spasticity/rigidity (n = 12/15), hyperactive tendon reflex (n = 11/14), pathological reflexes (n = 8/11), seizures (n = 9/16), dysphagia (n = 9/12), developmental delay (n = 7/14), infantile hypotonia (n = 3/11), and optic nerve atrophy (n = 2/7). Skeletal deformities were observed in 13/17 cases and fell within the osteopetrosis spectrum. One case had enamel abnormalities, whereas the remaining cases with sufficient data (n = 6) had normally developed teeth.

Table 4 provides the neuroimaging characteristics of the patients with BANDDOS. Brain abnormalities were present in all cases (n = 19) on neuroimaging, including white matter changes (n = 19/19), calcifications (n = 15/18), agenesis of corpus callosum (n = 12/16), ventriculomegaly (n = 13/19), Dandy-Walker complex (n = 7/19), and cortical abnormalities (n = 4/10).

**Table 4:** Neuroimaging characteristics of the patients with BANDDOS

No.	Dandy-Walker malformation (n = 7/19)	Ventriculomegaly (n = 13/19)	Calcifications (n = 15/18)	Agenesis of corpus callosum (n = 12/16)	White matter abnormalities (n = 19/19)	Cortical abnormalities (n = 4/10)
1	Yes	Supratentorial	Yes	Yes	Yes	Yes
2	Yes	Supratentorial	Yes	Yes	Yes	Yes
3	Yes	Yes	Yes	Yes	Yes	N/R
4	No	Yes	Yes	Yes	Yes	Yes
5	Large cysterna magna	Yes	Yes	No	Yes	N/R
6	Large cysterna magna	Yes	Yes	Yes	Yes	N/R
7	No	Yes	Yes	N/R	Yes	N/R
8	No	Yes	Yes	N/R	Yes	N/R
9	No	Yes	Yes	N/R	Yes	N/R
10	Yes	Yes	Yes	Yes	Yes	Yes
11	Yes	Yes	Yes	Yes	Yes	N/R
12	No	Yes	Yes	Yes	Yes	N/R
13	No	Yes	Yes	Yes	Yes	N/R
14	No	No	N/R	Yes	Yes	No
15	No	No	Yes	Yes	Yes	No
16	No	No	Yes	Yes	Yes	No
17	No	No	No	No	Yes	No
18	No	No	No	No	Yes	No
19	No	No	No	No	Yes	No

N/A = non-applicable; N/R = not reported;

Three patients died in infancy, two in childhood and one case at unspecified age, and a brain autopsy was performed in one case. Postmortem examination showed multiple brain anomalies, including an absence of corpus callosum, reduced volume of white matter, Dandy-Walker malformation, colpocephaly, numerous periventricular and brainstem calcifications, and heterotopia, abnormal gyration of hippocampi, and non-decussation of small pyramidal tracts [14]. Histological evaluation evidenced severe microglia deficiency with only rare spotting of abnormal microglia around the blood vessels, prominent white matter atrophy with axonal spheroids, gliosis, and numerous dystrophic calcifications predominantly in the periventricular white matter [14].

## Discussion

**Fig. 5:**
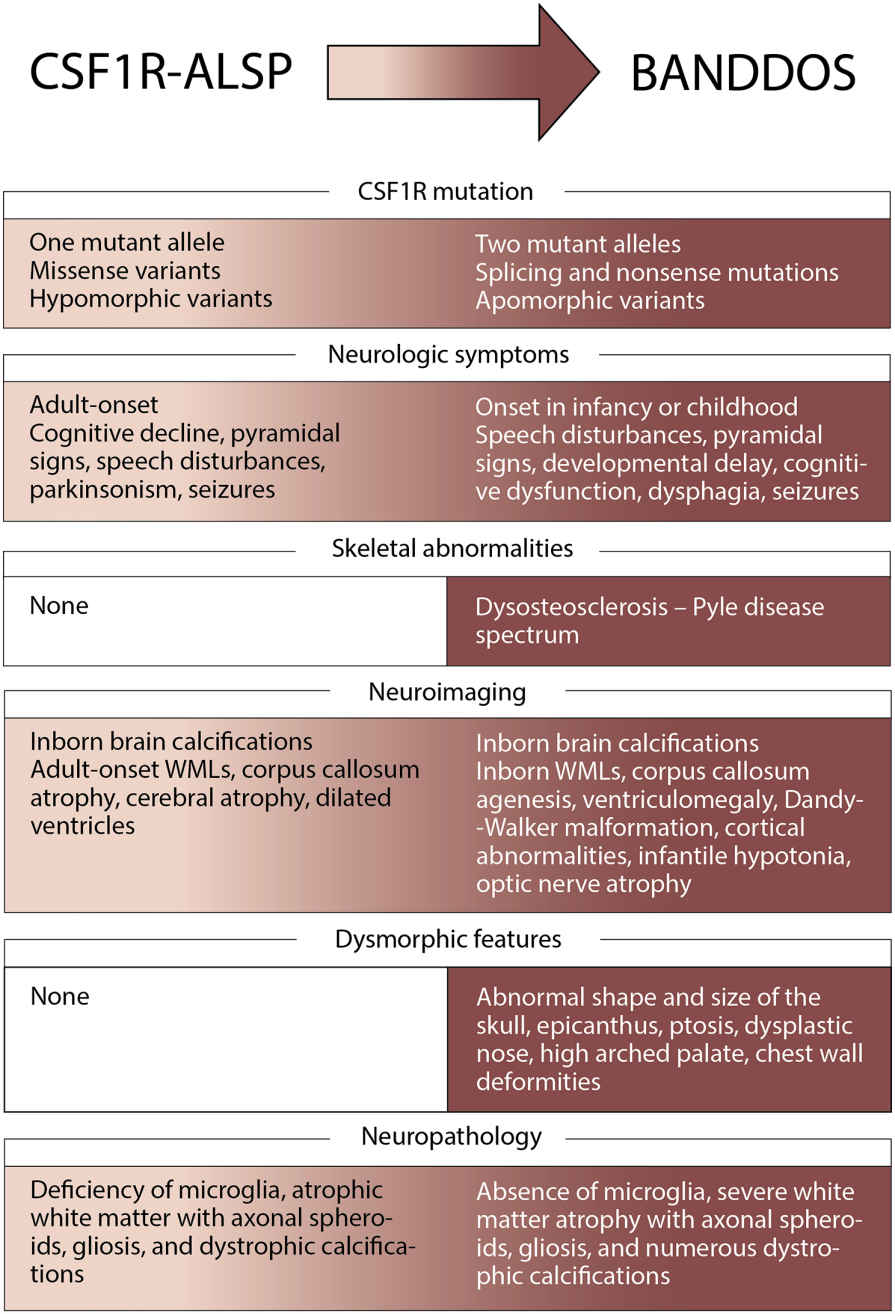
Schematic diagram of core features of the CSF1R-related adult-onset leukoencephalopathy with axonal spheroids and pigmented glia (CSF1R-ALSP) and brain abnormalities, neurodegeneration, and dysosteosclerosis (BANDDOS). Both disorders are on the same continuum, albeit differ in the severity of CSF1R mutations’ consequences and phenotypes. WMLs – white matter lesions

In this study, we compiled data on the largest number of BANDDOS cases to date. Although it is an exceedingly rare disease, it was reported in different parts of the world, encompassing Asia and South and North America. Most cases were born of consanguineous marriages after an uncomplicated pregnancy, with normal weight and length. Figure 5 presents and compares the core features of BANNDOS and *CSF1R*-ALSP.

Brain abnormalities were found in all cases and ranged from mild asymptomatic white matter changes to severe brain malformations. Similar to *CSF1R*-ALSP, white matter lesions were the most common finding on neuroimaging (in 100% of BANDDOS vs. 81% with *CSF1R*-ALSP cases), followed by calcifications (in 83% of BANDDOS vs. 75% with *CSF1R*-ALSP cases) and callosal abnormalities (agenesis in 75% of BANDDOS vs. atrophy in 29% with *CSF1R*-ALSP cases) [18]. Interestingly, calcifications in both disorders were present already at birth and shared a characteristic “stepping stone appearance” [2]. White matter lesions and callosal abnormalities were also present at birth in BANDDOS cases but were observed later in *CSF1R*-ALSP, in which they were seen around the time of the symptomatic disease onset in adulthood [2, 19]. Compared to *CSF1R*-ALSP, cases with BANDDOS also displayed other congenital brain anomalies, including Dandy-Walker malformation, ventriculomegaly, and cortical abnormalities.

BANDDOS and *CSF1R*-ALSP mainly manifest with neurological deficits. However, in the former first symptoms were most often observed in the first weeks of life, and developmental delay was present in half of the cases, whereas in the latter, the first three decades of life were usually unremarkable, and the disease started in the 4th -6th decade of life [3]. The earlier age of disease onset with the frequent developmental delay reflects the greater severity of the disease in BANDDOS. Speech disturbances were the most common neurological symptom in BANDDOS and were present in 87% of cases, with dysarthria reported in more than half of cases. Speech disorders are frequently observed in *CSF1R*-ALSP with complex underpinnings involving language disturbances (aphasia in up to 42%), articulation disorders (dysarthria in up to 54%), and not infrequently, both. As all cases with BANDDOS with speech disturbances had accompanying cognitive decline or developmental delay, the multifaceted nature of speech dysfunction, as seen in *CSF1R*-ALSP, is most likely. Progressive cognitive dysfunction was present in 86% of BANDDOS cases and was not reported only in two clinically asymptomatic Brazilian cases. Likewise, cognitive impairment is frequent in *CSF1R*-ALSP and occurs in 94% of cases [3]. Pyramidal signs (spasticity, hyperactive tendon reflexes, and pathological reflexes) were present in up to 80% of BANDDOS cases, compared to up to 81% of *CSF1R*-ALSP cases [3]. Seizures were noted in 56% of cases in BANDDOS, compared to up to 32% of *CSF1R*-ALSP [3]. Dysphagia was common and reported in 75% of cases of BANDDOS, compared to 18% of *CSF1R*-ALSP [3]. As seizures usually reflect more widespread and severe brain injury, and dysphagia is observed in the later disease stages in *CSF1R*-ALSP, the higher reported frequency of these symptoms in BANDDOS may be attributed to a more devastating disease course. In addition, a minority of BANDDOS cases presented infantile hypotonia (27%) and atrophy of the optic nerve (29%), which were not reported in *CSF1R*-ALSP. Skeletal abnormalities were observed in 76% of cases of BANDDOS. However, the extent of skeletal involvement and presentation was variable and generally fell within the dysosteosclerosis – Pyle disease spectrum. Dysmorphic features were reported in 41% of cases of BANDDOS. Skeletal deformities and dysmorphic features were often associated with complex brain abnormalities and a more ominous clinical course; however, they were also observed in one case with the adult onset of the disease. Skeletal deformities and dysmorphic features have not been reported in *CSF1R*-ALSP. Therefore, the presence thereof may reflect more profound sequelae of the *CSF1R* mutations in the BANDDOS, which impacted organs beyond the central nervous system. Similar to other childhood-onset leukoencephalopathies, the quality of life of the patients and their caregivers is severely compromised [20–22].

Neuropathological evaluation of one case with BANDDOS evidenced significant overlap with *CSF1R*-ALSP [2, 9, 14]. Macroscopically both disorders shared extensive white matter degeneration, most prominent in the periventricular region, corpus callosum, and pyramidal tracts [2, 9, 14]. Likewise, histological evaluation evidenced a deficiency of microglia in the brain parenchyma, atrophic white matter with axonal spheroids, gliosis, and dystrophic calcifications in BANDDOS and *CSF1R*-ALSP [2, 9, 14]. However, the former had much greater severity of the disease process, as reflected by macroscopic (absence of corpus callosum, Dandy-Walker malformation, and other) and microscopic (almost complete absence of microglia) findings [14].

The CSF1R is a transmembrane tyrosine-protein kinase serving as a receptor for CSF1 (colony-stimulating factor-1) and interleukin-34 (IL34) [1, 23, 24]. It participates in the innate immunity and inflammatory response through the release of pro-inflammatory cytokines [23]. It also plays a crucial role in the development, proliferation, activation, and survival of the monocyte phagocytic system [1, 23, 25, 26]. Both microglia of the central nervous system and osteoclasts stem from the monocyte phagocytic system, and their differentiation process depends on CSF1R. Therefore, a properly functioning CSF1R is required for the development and mainantence of the central nervous system, bone and teeth, as well as, immune system. It was also demonstrated that CSF1R is important for developing milk ducts, acinar structures, and both female and male reproductive tracts [23].

The CSF1R protein consists of 972 amino acids, including regulatory juxtamembrane domain (amino acids 542–574), tyrosine kinase domain (amino acids 582–910), and activation loop domain (amino acids 796–818) [23]. Both juxtamembrane and activation loop domains have regulatory functions, and phosphorylation thereof induces conformational changes and activates the tyrosine kinase domain [23]. In *CSF1R*-ALSP, more than 106 pathogenic mutations in *CSF1R* were identified [1]. Similar to *CSF1R*-ALSP, most of the identified *CSF1R* mutations in BANDDOS (n = 9/11) affected the tyrosine kinase domain; however, severe variants were much more common in the letter. In the *CSF1R*-ALSP, almost 80% of variants are missense mutations [3]. Although the genotype-phenotype relationships in *CSF1R*-ALSP are not well understood, patients with variants causing CSF1R protein truncation or nonsense-mediated mRNA decay were shown to have an earlier age of onset [1]. In BANDDOS, missense variants accounted for less than a third of all mutations. In one case with a missense variant outside the mutational “hot spot” (Pro132Leu), there was a protein-truncating variant (Gln481*) on the other allele leading to the CSF1R protein completely devoid of the tyrosine kinase domain. Furthermore, in BANDDOS, the splicing and nonsense variants leading to a severe loss of CSF1R protein function were the most common type of mutation.

To date, no identical mutation has been reported to occur in both disorders. In 2019 Oosterhof et al. [14] reported a patient with a bi-allelic Gly585_Lys619delinsAla mutation which had been previously reported in *CSF1R*-ALSP by Radamekers et al. [27]; however, the mutations were not identical on the coding level. Different substitution mutation at the same base pair position was reported in BANDDOS, c.2498 C > T [16], and in *CSF1R*-ALSP, c.2498 C > A [28, 29]. Therefore, we think the apparent “dichotomy” in mutations between *CSF1R*-ASLP in BANDDOS is due to the rarity of the disorders rather than different pathomechanisms. As the genetic testing becomes more readily available and *CSF1R* mutations are increasingly recognize worldwide, we predict the genetic landscapes of both disorders will soon be bridged [1, 3, 4, 30–32].

BANDDOS and CSF1R-ALSP share a genetic basis and a significant overlap of clinical, radiological, and pathological features. Therefore, both disorders are on the same continuum, albeit they differ in the severity of CSF1R mutations’ consequences and phenotypes. This opens windows of opportunity for applying already available therapy in *CSF1R*-ALSP in BANDDOS. Bone marrow transplant (BMT) was shown to be effective CSF1R-ALSP [33, 34]. Another clinical trial with a TREM2 agonist, which could rescue or compensate for *CSF1R* protein deficiency, is underway (NCT05677659) [1]. In addition, CSF1R protein was shown to play a role in other neurodegenerative diseases, and its inhibitors showed promising results in preclinical studies [35]. Therefore, further studies on *CSF1R*-related disorders may lead to a better understanding of these disorders and pave the way toward curative therapy in other neurological disorders.

The main limitation of the present study is the relatively low number of cases included into the analsysis. However, this is due to the rarity of the BANDDOS and the present paper presents the largest analysis on patients with BANDDOS to date. Secondly, the patients carried different *CSF1R* mutations, occasionally in the compund heterozygous state, and it was not possible to determine genotype-phenotype correlations. As the genetic testing with NGS is becoming more readily available, we speculate that more patients will be identified with variants of unknown significance within *CSF1R* gene, in isolation or in combination with other known *CSF1R* pathogenic mutations. Therefore, more research is needed to determine the pathophysiology of *CSF1R*-related neurodegeneration and genotype-phenotype correlations.

## Conclusions

BANDDOS is an exceedingly rare disorder with a wide spectrum of the age of onset and disease severity; however, in most cases, it starts in the perinatal period or infancy and has a devastating course with congenital brain abnormalities, developmental delay, neurological deficits, osteopetrosis, and dysmorphic features. There is a significant overlap in the clinical, radiological, and neuropathological aspects between BANDDOS and CSF1R-ALSP. Both disorders share a genetic basis and are manifestations of CSF1R protein deficiency, albeit of different extents of disease severity. Therefore is a window of opportunity for translating treatment from *CSF1R*-ALSP to BANDDOS.

## Data Availability

Data available on request from the corresponding author.

## List of Abbreviations

BANDDOS: Brain abnormalities, neurodegeneration, and dysosteosclerosis
CSF1R: Colony-stimulating factor-1 receptor
CSF1R-ALSP: CSF1R-related leukoencephalopathy with axonal spheroids and pigmented glia
